# Temperature-responsive release of thyroxine and its environmental adaptation in Australians

**DOI:** 10.1098/rspb.2013.2747

**Published:** 2014-03-22

**Authors:** Xiaoqiang Qi, Wee Lee Chan, Randy J. Read, Aiwu Zhou, Robin W. Carrell

**Affiliations:** 1 Department of Haematology, Cambridge Institute for Medical Research, University of Cambridge, Wellcome Trust/MRC Building, Hills Road, Cambridge CB2 0XY, UK; 2 Key Laboratory of Cell Differentiation and Apoptosis of Ministry of Education of China, School of Medicine, Shanghai JiaoTong University, No. 280, Shanghai 200025, People's Republic of China

**Keywords:** thyroxine, thyroxine-binding globulin, aboriginal Australian, febrile convulsions, hypothermia, hibernation

## Abstract

The hormone thyroxine that regulates mammalian metabolism is carried and stored in the blood by thyroxine-binding globulin (TBG). We demonstrate here that the release of thyroxine from TBG occurs by a temperature-sensitive mechanism and show how this will provide a homoeostatic adjustment of the concentration of thyroxine to match metabolic needs, as with the hypothermia and torpor of small animals. In humans, a rise in temperature, as in infections, will trigger an accelerated release of thyroxine, resulting in a predictable 23% increase in the concentration of free thyroxine at 39°C. The *in vivo* relevance of this fever-response is affirmed in an environmental adaptation in aboriginal Australians. We show how two mutations incorporated in their TBG interact in a way that will halve the surge in thyroxine release, and hence the boost in metabolic rate that would otherwise occur as body temperatures exceed 37°C. The overall findings open insights into physiological changes that accompany variations in body temperature, as notably in fevers.

## 1. Introduction

Thyroxine is the hormone that most directly controls mammalian activity, with its immediate derivatives regulating cellular oxygen consumption and the metabolism of body and brain [1]. Consequently, tissue concentrations of thyroxine are precisely defined: too much leads to hyperactivity and too little to dormancy. The storage and transport of thyroxine in blood has been well documented in humans [2,3]. The steady-state concentration of thyroxine in blood is set centrally by the secretion of thyroid stimulating hormone (TSH) but the maintenance of this concentration throughout the tissues is owing to the equilibrated release of thyroxine from its carrier protein in the blood, thyroxine-binding globulin (TBG) [2–4]. The binding affinity of TBG for thyroxine is exceptionally tight such that in humans only 0.03% of the total blood thyroxine is in the free form, at picomolar concentration. Other thyroxine carriers in blood, albumin and transthyretin, contribute to the equilibration of plasma concentrations but their influence is minor [5]. The overwhelming proportion of the circulating thyroxine is bound as a ligand to TBG, which acts as both a store and a buffer to give an equilibrated release of thyroxine to the tissues. The binding capacity of circulating TBG is only partially saturated, and it is the percentage saturation, 20% or more, that by the law of mass action determines the concentration of free thyroxine in the tissues. Although the steady-state concentration of free thyroxine and hence the percentage saturation of TBG is determined centrally we demonstrate here, as we have similarly shown with the closely related corticosteroid-binding globulin (CBG) [6,7], how the release of thyroxine from TBG is further adjusted within the circulation by a temperature-sensitive modulation of its binding affinity.

**Figure 1.**
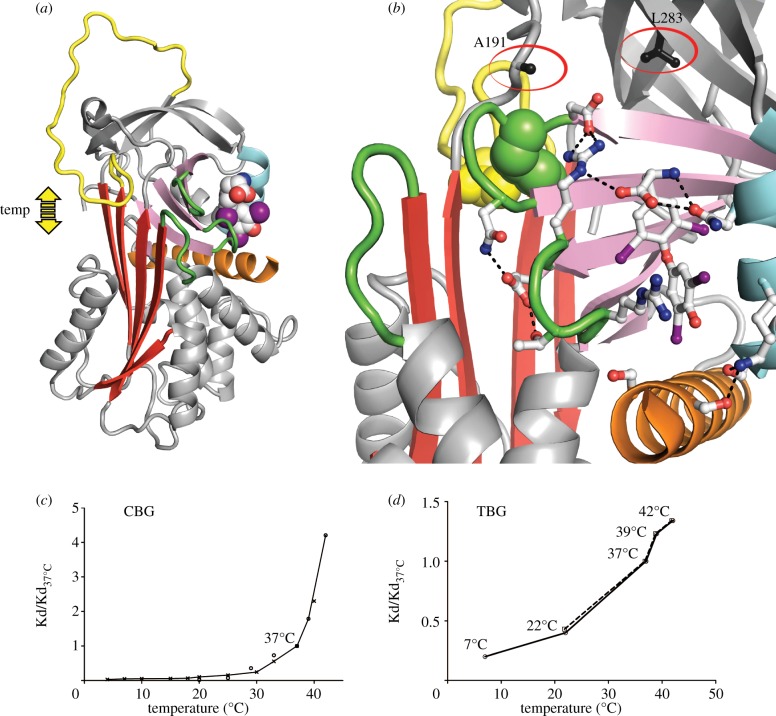
Temperature-responsive release of thyroxine from TBG. (*a*) Thyroxine, in space-filling form. Movement of the reactive centre loop (yellow) into and out of the A-sheet (red) of TBG directly affects the binding site magnified in (*b*) showing the interactions that stabilize thyroxine (skeletal form) in the binding site. Entry of the reactive centre loop will cause a steric perturbation and the expansion of the A-sheet will displace the connecting loops (green) that surround the bound thyroxine. The Australian mutations, A191T and L283F, flank the binding site. (*c*,*d*) The proportional loss of hormone-binding affinity with increasing temperature (Kd/Kd_37°C_): (*c*) shown with the homologous CBG, from Chan [7] circles and Mickelson [14] crosses; (*d*) with TBG and fluorophore–thyroxine data from table 1. The plot of the L283F variant of TBG (interrupted line) is superimposable on that of the wild-type, including the inflection at 37°C.

TBG and CBG are members of the serpin family of serine protease inhibitors, with homologous hormone-binding sites. Although both hormone carriers have lost any inhibitory activity, they retain the ability to undergo the remarkable conformational change, from a stressed to a relaxed form, that characterizes the serpins [8,9]. Recent structure-based studies [4,10–13] show how the initiating stage in this change, the temperature-sensitive movement of the reactive loop into and out of the main beta-sheet of the molecule, affects the plasticity of the underlying hormone-binding pocket (figure 1*a*,*b*). When the reactive loop is fully exposed, as favoured at lower temperatures, the binding pocket readily adopts an optimal conformation with a high binding affinity. Conversely, the partial entry of the reactive loop into the A-sheet as the temperature rises will perturb the binding pocket with a resultant decrease in affinity and increased release of the bound hormone.

The existence of this temperature-responsive mechanism [15] is readily demonstrable with CBG [6,7], which has a measurable change in fluorescence on release of its bound cortisol (figure 1*c*). The assessment of changes in the affinity of TBG, however, is more challenging, as it undergoes only minor shifts in fluorescence, with the monitoring of its dissociation rate being further complicated by its exceptionally strong binding affinity (Kd 80 pM). To overcome this, we synthesized [13] a fluorescent thyroxine-adduct that is structurally identical to thyroxine in its binding to TBG but does so with a much-decreased affinity.

## 2. Material and methods

Wild-type recombinant human TBG and its engineered variants, A191T, L283F and A191T/L283F and the fluorophore-adduct of thyroxine (l-thyroxine-6-carboxyfluoroscein) were prepared as previously described [4,13]. Binding affinities were determined by fluorescence titration in phosphate buffer pH 7.4 at defined temperatures as detailed in Qi *et al*. [13]. All Kd measurements were carried out three times in duplicate. Thermal stability of recombinant variants was determined by ThermoFluor assay: samples were heated gradually to denaturation; the exposure of the hydrophobic core allowing the dye, SYPRO Orange, to bind and fluoresce. Figure 1*a*,*b* was prepared from our previously deposited coordinates as detailed in Zhou *et al*. [12].

Thyroxine indices: free thyroxine and % saturation of TBG and its variants over a range of temperatures were calculated using a free-thyroxine concentration at 37°C of 20 pM based, with recent updating [16], on a plasma range of 12–26 pM. The binding constant Kd of thyroxine with TBG at 37°C and pH 7.4 has been variously reported (bracketed, inverse Ka ×10(10) M-1): in 1972 [17] as 60 pM (Ka 1.68), more definitively with isolated TBG by Korcek & Tabachnik [18] in 1976 as 110 pM (Ka 0.90), in plasma by Ross & Benraad [19] 1992 as 67 pM (Ka 1.5) and from our own competitive-assay with recombinant TBG [13] as 75 pM (Ka 1.33). We have adopted here the mean of these results, a Kd of 80 pM (Ka 1.25), but note that whatever affinity is chosen will affect only the magnitude of derived values and not the proportional changes central to this paper. With a Kd of 80 pM and a free thyroxine at 37°C of 20 pM, the saturation of TBG will be 20%.

Other proteins that bind thyroxine in the plasma do so with a very much lower affinity than TBG. As Schreiber describes [5] and others show experimentally [19,20], if the concentration of free thyroxine is less than the Kd of TBG, as in plasma, the changes in free thyroxine can be directly derived from the changes in the Kd of TBG. This allows the calculation here of the concentration of free thyroxine [FT4] and the percentage saturation of TBG, Y, from the law of mass action:
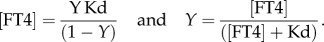


## 3. Results and discussion

The thyroxine–fluorophore was used to determine the proportional change in binding affinity, Kd/Kd_37°C_ that takes place over a range of temperatures in human TBG and its engineered variants (table 1*b*).

**Table 1.** Variation of TBG binding affinities and free-thyroxine concentrations with temperature. (*a*) Kd, and Kd/Kd_37°C_ ratios of recombinant TBG variants with the thyroxine–fluorophore. Kd measurements were repeated more than three times in duplicate; data are means ± s.d. (*b*) The fluorophore Kd/Kd_37°C_ derived changes in free thyroxine (FT4) with temperature (shown in bold) are in consistent agreement with previous independently derived values with thyroxine and isolated plasma TBG [20] (non-bold), and the assay of free thyroxine in plasma at 21°C and 37°C, [19,20] (figure 2). Calculations based on FT4 of 20 pM and TBG saturation of 20% at 37°C.

(*a*) TBG: thyroxine–fluorophore binding affinity with temperature
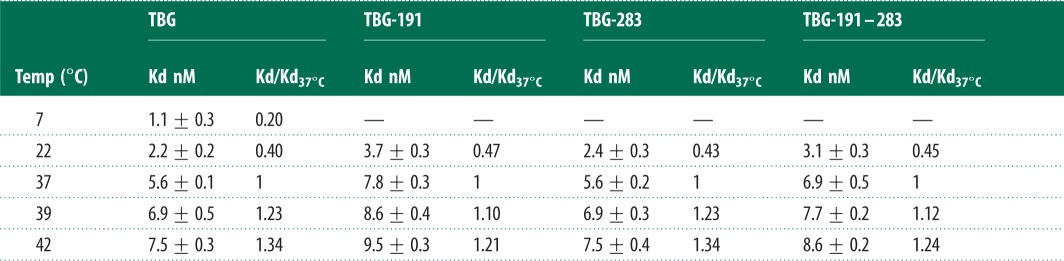
(*b*) Free thyroxine (FT_4_) from fluorophore ratios and direct T_4_ Kd values
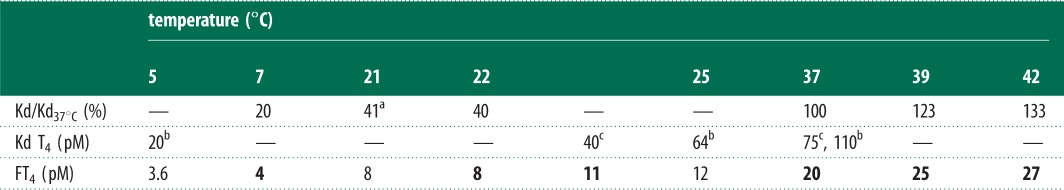

^a^Serum FT_4_ 21°C/37°C ratio, Ross & Benraad [19].

Kd T_4_: ^b^Korcek *et al*. [18], ^c^Qi *et al*. [13].

**Figure 2.**
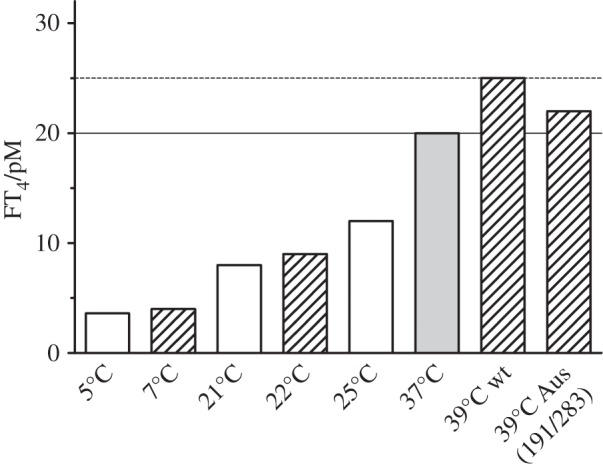
Variation in free thyroxine with temperature from Kd/Kd_37°C_ ratios (hatched bars), 20 pM at 37°C rising to 25 pM at 39°C with wt-TBG, but dampened to 22 pM in the 191/283 TBG (Aus) variant (upper normal limit, dashed line). The open (non-hatched) bars show values from independent determinations by others using plasma-derived thyroxine [18] and direct assays of plasma-free thyroxine [19,20]. The comparative bars are based on a defined Kd of 80 pM, free thyroxine of 20 pM and a TBG saturation of 20%, at 37°C.

The use of the fluorophore–thyroxine Kd/Kd_37°C_ ratios to calculate the proportional changes that will occur to the binding affinity of thyroxine to TBG in plasma is validated in table 1*a* and figure 2 by the agreement with values independently determined with thyroxine [18] and with the direct assays by others of free-thyroxine concentrations in the blood at room and body temperature [19,20]. The plot of Kd/Kd_37°C_ ratios versus temperature in figure 1*d* and of consequent blood free-thyroxine concentrations (table 1*b* and figure 2) demonstrate how changes in the binding affinity of TBG provide an inherent adjustment of thyroxine levels to match metabolic needs.

### (a) Small mammals and hypothermia

Although our data here and almost all the detailed knowledge of the physiology of thyroxine transport are derived from the human, there is clear evidence that the system is strongly conserved in all mammals. This is seen not only in the conservation of TBG sequence in diverse mammals [21] but also in the identity of molecular mechanisms based on crystal structures from the mouse [11] and the human [12,13]. Hence, the findings here (figures 1*d* and 2) have direct relevance to the hypothermia and torpor that occur in small animals [22,23]. Based on a saturation of TBG in the human of 20%, there will be a homoeostatic decrease in the concentration of free thyroxine as body temperature falls, with a fivefold drop from 20 pM at 37°C to a baseline 4 pM at 7°C. Similarly, as body temperature is restored, the thyroxine, stably stored in the TBG, will be increasingly released, rising to meet the needs of full activity at 37°C.

### (b) Humans and fever

The critical metabolic demands of a much larger brain make humans especially sensitive to changes in the release of thyroxine and even mild hypothermia, if prolonged, is fatal. A physiological change in body temperature does, however, occur in humans with the fevers that are induced by inflammation and infection. The accelerated decrease in binding affinity that will accompany the increase in body temperature from 37° to 39°C and, exceptionally, to 42°C is seen in figure 1*d*. Evidence that this fever-induced decrease in binding affinity is specific and purposeful and comes from a similar but even greater acceleration of hormone release in the closely related CBG (figure 1*c*). The loss of affinity in TBG at fever temperatures has, however, even more direct physiological impact than that of CBG because of the much tighter hormone-binding affinity of TBG and the precisely defined limits of free-thyroxine concentration in the blood. Any changes in the Kd of TBG will be directly reflected in changes in free-thyroxine levels in the blood with a rise in body temperature to 39°C predictably giving a 23% increase in concentration, temporarily moving into the range seen in the clinical disorder of hyperthyroidism (figure 2).

### (c) Adaptation in the aboriginal Australian

**Figure 3.**
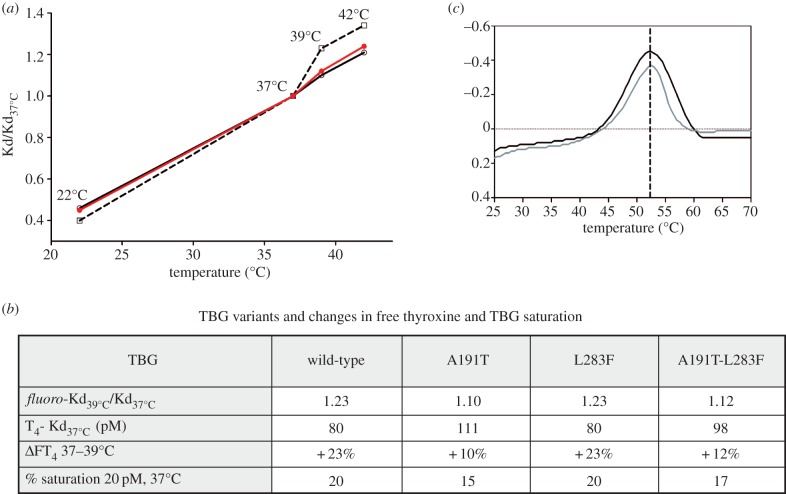
Changed binding affinity of Australian variant TBG at raised body temperatures. (*a*) Modified response of the A191T variant (full line) and the double A191T/L283F variant (red) compared with wild-type TBG (interrupted line). (*b*) Percentage increase in free thyroxine (ΔFT4) at 39°C and percent-saturation of each variant needed to give a free thyroxine of 20 pM at 37°C, calculated from Kd_39°C_/Kd_37°C_ ratio and derived thyroxine affinity (T4-Kd_37°C_) from table 1*a*. (*c*). As with the plasma variant [27,28], the recombinant A191/L283F TBG (black) has a small diminution in thermal stability to 52°C, compared with the wild-type 55°C [9]. The identical change in the single A191 recombinant (grey) confirms that the instability is independent of the L283F mutation.

The *in vivo* relevance of this surge in thyroxine release in fevers is affirmed by what had been a perplexing finding, the presence of two linked mutations in the TBG of aboriginal Australians [24–26]. Surveys in West Australia had shown the presence of this variant TBG in some 40% of the aboriginal population in association with lowered levels of total-thyroxine and total-TBG. The clue as to the functional significance of the mutations, a replacement of alanine 191 by a threonine and of leucine 283 by a phenylalanine, came from their placement on the periphery of the thyroxine-binding site [4] (figure 1*b*). Alanine 191 is immediately adjacent to the point of entry of the reactive loop into the main beta-sheet of TBG and its replacement by a polar threonine will predictably affect the H-bond network that links to the bound thyroxine. The consequence of this replacement is shown here with the change in thyroxine-binding affinity of the recombinantly expressed Ala191Thr TBG. The replacement critically results (figure 3*a*,*b*) in an abolition of the accelerated release of thyroxine that takes place as the temperature rises above 37°C, with the 23% increase in free thyroxine that would otherwise occur at 39°C being reduced by the mutation to a 10% increase.

An enigmatic accompanying finding strengthens the deduction that the Ala191Thr mutation in aboriginal Australians is selectively advantageous. This is the presence of the linked second mutation, with the replacement of the leucine at 283 by a bulkier phenylalanine. The puzzle was that this Leu283Phe mutation occurs not uncommonly in other populations without any discernible functional consequences [26]. We confirm here (table 1 and figure 1*d*) that this variant does, by itself, precisely retain the affinity and temperature response of the wild-type TBG. However, when the Leu283Phe replacement is co-expressed with that of Ala191Thr there is clearly an advantageous interaction. The thyroxine affinity of the double mutant moves closer to the physiological Kd at 37°C, with little loss of the thermal protection provided by the Ala191Thr replacement. In doing so, the linked mutation maintains the percentage saturation of TBG closer to the norm. With just the single Ala191Thr mutation, the saturation of TBG would need, by the law of mass action, to decrease from 20 to 15% in order to provide a physiological free thyroxine of 20 pM at 37°C. But with the addition of the second Leu283Phe mutation, this decrease changes to a more adequate 17% saturation.

The findings fit well with the quantitation by Takamatsu *et al.* in 1987 [27] of ancillary changes in an aboriginal hemizygote for the A191T/L283F TBG, giving a TBG concentration 74% of that of the normal and a much-reduced total thyroxine at 58% of a normal pool. The decreased overall level to 74% is in keeping with studies of other serpins, which show that functional mutations consistently result in a comparable decrease in the efficiency of expression. The further reduction in the saturation of this diminished level of TBG, as determined here with the recombinant variant from 20 to 17% (figure 3*b*), would result in an overall reduction in the total thyroxine to 62%, compatible with the 58% observed in the 1987 study. Further support for the relevance of the findings reported here with recombinant TBG to those occurring *in vivo* [27] is provided by the identical denaturation temperature of the recombinant double mutant at 52°C (figure 3*c*) to that measured in an earlier study of the plasma variant [28] and, as in that study, being only slightly decreased from the denaturation temperature of the wild-type plasma TBG at 55°C [8].

Thus, the paired polymorphisms provide the aboriginal Australian with a TBG that maintains its properties as a storage and carrier protein while having the additional local advantage of providing a reduced metabolic response to increased body temperatures*.* In a temperate climate, the boost to thyroxine release and increased metabolism that accompanies the rise in body temperatures in fevers will be an advantageous response to infection. But the same accelerated increase in metabolism could affect the survival of a population historically exposed to the arid environment of central Australia, with ambient temperatures of 45°C or above. There the life threatening risk is not so much the infection itself, but rather the dehydration and heat exhaustion that accompany dysentery and other common illnesses in infancy and childhood.

### (d) Wider implications

The recognition of this environmental adaptation in the aboriginal Australian has much wider significance in affirming the physiological relevance of the temperature-regulated release of thyroxine in blood. Although the central secretion of TSH controls the concentration of thyroxine in the longer term, the variations with temperature of free-thyroxine concentrations in the circulation will be rapid and reversible. This temperature responsive adjustment of the concentration of free thyroxine has been largely overlooked in the past, as it had been assumed that the thyroxine–TBG binding affinity remains constant. Moreover, thyroxine assays have customarily been carried out at room temperature. Measured retrospectively in this way, blood samples taken in hypothermia, in heatstroke, or from an infant with fever, will all be reported as having an unchanged free thyroxine.

The demonstration that TBG, as with CBG [6,7], acts as a protein thermocouple, has direct physiological implications. Such temperature-sensitive regulation of hormone release will affect everyday lives. For example, the accelerated release that will take place as the body core temperature rises to 39°C in a hot-bath or sauna will contribute to an enhancement of the metabolism of body and mind—euphoria and eureka! A similar boost in hormone release also opens a contributory explanation for the common occurrence of febrile convulsions in infancy [29]. The brain is sensitive to changes in free thyroxine and raised levels in thyrotoxicosis in adults can result in convulsive seizures that cease, with no after-effects, when the thyroxine level returns to normal [30,31]. Comparable seizures also commonly occur in infants in conjunction with the spiking increases in temperature that accompany incidental infections at that age. The surge in thyroxine release that will occur in response to the increases in the temperature of the brain in fevers [32] poses a potential exacerbating factor in the childhood seizures—a conclusion reinforced by the prompt cessation of the convulsions as the infant's body temperature is cooled.

## References

[1] Cheng SY Leonard JL Davis PJ 2010 Molecular aspects of thyroid hormone actions. Endocr. Rev. 31, 139–170 (doi:10.1210/er.2009-0007) 20051527 10.1210/er.2009-0007 PMC2852208

[2] Robbins J 2000 Editorial: new ideas in thyroxine-binding globulin biology. J. Clin. Endocrinol. Metab. 85, 3994–3995 11095420 10.1210/jcem.85.11.7049

[3] Refetoff S 2014 Thyroid hormone serum transport proteins: structure, properties, genes and transcriptional regulation. South Dartmouth, MA: Endocrine Education Inc. See http://www.thyroidmanager.org

[4] Zhou A Wei Z Read RJ Carrell RW 2006 Structural mechanism for the carriage and release of thyroxine in the blood. Proc. Natl Acad. Sci. USA 103, 13 321–13 326 (doi:10.1073/pnas.0604080103) 10.1073/pnas.0604080103 PMC1557382 16938877

[5] Schreiber G 2002 The evolutionary and integrative roles of transthyretin in thyroid hormone homeostasis. J. Endocrinol. 175, 61–73 (doi:10.1677/joe.0.1750061) 12379491 10.1677/joe.0.1750061

[6] Cameron A Henley D Carrell R Zhou A Clarke A Lightman S 2010 Temperature-responsive release of cortisol from its binding globulin: a protein thermocouple. J. Clin. Endocrinol. Metab. 95, 4689–4695 (doi:10.1210/jc.2010-0942) 20631013 10.1210/jc.2010-0942

[7] Chan WL Carrell RW Zhou A Read RJ 2013 How changes in affinity of corticosteroid-binding globulin modulate free cortisol concentration. J. Clin. Endocrinol. Metab. 98, 3315–3322 (doi:10.1210/jc.2012-4280) 23783094 10.1210/jc.2012-4280 PMC3813945

[8] Pemberton PA Stein PE Pepys MB Potter JM Carrell RW 1988 Hormone binding globulins undergo serpin conformational change in inflammation. Nature 336, 257–258 (doi:10.1038/336257a0) 3143075 10.1038/336257a0

[9] Huber R Carrell RW 1989 Implications of the three-dimensional structure of alpha 1-antitrypsin for the structure and function of serpins. Biochemistry 28, 8951–8966 (doi:10.1021/bi00449a001) 2690952 10.1021/bi00449a001

[10] Grasberger H Golcher HM Fingerhut A Janssen OE 2002 Loop variants of the serpin thyroxine-binding globulin: implications for hormone release upon limited proteolysis. Biochem. J. 365, 311–316 (doi:10.1042/BJ20020014) 11931635 10.1042/BJ20020014 PMC1222644

[11] Klieber MA Underhill C Hammond GL Muller YA 2007 Corticosteroid-binding globulin, a structural basis for steroid transport and proteinase-triggered release. J. Biol. Chem. 282, 29 594–29 603 (doi:10.1074/jbc.M705014200) 17644521 10.1074/jbc.M705014200

[12] Zhou A Wei Z Stanley PL Read RJ Stein PE Carrell RW 2008 The S-to-R transition of corticosteroid-binding globulin and the mechanism of hormone release. J. Mol. Biol. 380, 244–251 (doi:10.1016/j.jmb.2008.05.012) 18513745 10.1016/j.jmb.2008.05.012

[13] Qi X 2011 Allosteric modulation of hormone release from thyroxine and corticosteroid-binding globulins. J. Biol. Chem. 286, 16 163–16 173 (doi:10.1074/jbc.M110.171082) 10.1074/jbc.M110.171082 PMC3091225 21325280

[14] Mickelson KE Harding GB Forsthoefel M Westphal U 1982 Steroid-protein interactions. Human corticosteroid-binding globulin: characterization of dimer and electrophoretic variants. Biochemistry 21, 654–660 (doi:10.1021/bi00533a010) 7074030 10.1021/bi00533a010

[15] Beauchamp NJ 1998 Antithrombins Wibble and Wobble (T85M/K): archetypal conformational diseases with *in vivo* latent-transition, thrombosis, and heparin activation. Blood 92, 2696–2706 9763552

[16] Thienpont LM Van Uytfanghe K Van Houcke S Tests IWGfSoTF 2010 Standardization activities in the field of thyroid function tests: a status report. Clin. Chem. Lab. Med. 48, 1577–1583 (doi:10.1515/CCLM.2010.321) 21034252 10.1515/CCLM.2010.321

[17] Green AM Marshall JS Pensky J Stanbury JB 1972 Studies on human thyroxine-binding globulin. VII. The effect of environmental changes on the fluorescence of I,8-anilinonaphthalene sulfonic acid bound to thyroxine-binding globulin. Biochim. Biophys. Acta 278, 305–315 (doi:10.1016/0005-2795(72)90236-X) 4628533

[18] Korcek L Tabachnick M 1976 Thyroxine–protein interactions. Interaction of thyroxine and triiodothyronine with human thyroxine-binding globulin. J. Biol. Chem. 251, 3558–3562 6458

[19] Ross HA Benraad TJ 1992 Is free thyroxine accurately measurable at room temperature? Clin. Chem. 38, 880–886 1597014

[20] van der Sluijs Veer G Vermes I Bonte HA Hoorn RK. 1992 Temperature effects on free-thyroxine measurements: analytical and clinical consequences. Clin. Chem. 38, 1327–1331 1623599

[21] The UniProt Consortium 2013 Update on activities at the Universal Protein Resource (UniProt) in 2013. Nucleic Acids Res. 41, D43–D47 (doi:10.1093/nar/gks1068) 23161681 10.1093/nar/gks1068 PMC3531094

[22] Jallageas M Mas N Assenmacher I 1989 Further demonstration of the ambient temperature dependence of the annual biological cycles in the edible dormouse, *Glis glis*. J. Comp. Physiol. B 159, 333–338 (doi:10.1007/BF00691513) 2778130 10.1007/BF00691513

[23] Geiser F 2004 Metabolic rate and body temperature reduction during hibernation and daily torpor. Annu. Rev. Physiol. 66, 239–274 (doi:10.1146/annurev.physiol.66.032102.115105) 14977403 10.1146/annurev.physiol.66.032102.115105

[24] White GH Morice R 1980 Diagnostic biochemical tests in Aboriginals. Med. J. Aust. 1, 6–8 7393024 10.5694/j.1326-5377.1980.tb135259.x

[25] Dick M Watson F 1981 A possible variant of thyroxine-binding globulin in Australian Aborigines. Clin. Chim. Acta 116, 361–367 (doi:10.1016/0009-8981(81)90055-3) 6794959 10.1016/0009-8981(81)90055-3

[26] Takeda K 1989 Sequence of the variant thyroxine-binding globulin of Australian aborigines. Only one of two amino acid replacements is responsible for its altered properties. J. Clin. Invest. 83, 1344–1348 (doi:10.1172/JCI114021) 2495303 10.1172/JCI114021 PMC303827

[27] Takamatsu J Refetoff S Charbonneau M Dussault JH 1987 Two new inherited defects of the thyroxine-binding globulin (TBG) molecule presenting as partial TBG deficiency. J. Clin. Invest. 79, 833–840 (doi:10.1172/JCI112891) 3102557 10.1172/JCI112891 PMC424213

[28] Murata Y Refetoff S Sarne DH Dick M Watson F 1985 Variant thyroxine-binding globulin in serum of Australian aborigines: its physical, chemical and molecular properties*.* J. Endocrinol. Invest. 8, 225–232 3928734 10.1007/BF03348482

[29] Friderichsen C Melchior J 1954 Febrile convulsions in children, their frequency and prognosis. Acta Paediatr. 43 (Suppl.), 307–317 (doi:10.1111/j.1651-2227.1954.tb15480.x) 10.1111/j.1651-2227.1954.tb15480.x 13228037

[30] Jabbari B Huott AD 1980 Seizures in thyrotoxicosis. Epilepsia 21, 91–96 (doi:10.1111/j.1528-1157.1980.tb04048.x) 6766396 10.1111/j.1528-1157.1980.tb04048.x

[31] Song TJ Kim SJ Kim GS Choi YC Kim WJ 2010 The prevalence of thyrotoxicosis-related seizures. Thyroid 20, 955–958 (doi:10.1089/thy.2009.0276) 20718679 10.1089/thy.2009.0276

[32] Rossi S Zanier ER Mauri I Columbo A Stocchetti N 2001 Brain temperature, body core temperature, and intracranial pressure in acute cerebral damage. J. Neurol. Neurosurg. Psychiatry 71, 448–454 (doi:10.1136/jnnp.71.4.448) 11561026 10.1136/jnnp.71.4.448 PMC1763520

